# Heterogeneous Solid Electrolyte Interphase Interactions Dictate Interface Instability in Sodium Metal Electrodes

**DOI:** 10.1002/advs.202404887

**Published:** 2024-07-30

**Authors:** Aditya Singla, Kaustubh G. Naik, Bairav S. Vishnugopi, Partha P. Mukherjee

**Affiliations:** ^1^ School of Mechanical Engineering Purdue University West Lafayette IN 47907 USA

**Keywords:** electro‐chemo‐mechanical heterogeneities, filament growth, Na morphology, nonuniform stresses, SEI failure, solid electrolyte interphase

## Abstract

Sodium (Na) metal batteries have attracted recent attention due to their low cost and high abundance of Na. However, the advancement of Na metal batteries is impeded due to key challenges such as dendrite growth, solid electrolyte interphase (SEI) fracture, and low Coulombic efficiency. This study examines the coupled electro‐chemo‐mechanical interactions governing the electrodeposition stability and morphological evolution at the Na/electrolyte interface. The SEI heterogeneities influence transport and reaction kinetics leading to the formation of current and stress hotspots during Na plating. Further, it is demonstrated that the heterogeneity‐induced Na metal evolution and its influence on the stress distribution critically affect the mechanical overpotential, contributing to a faster SEI failure. The analysis reveals three distinct failure mechanisms—mechanical, transport, and kinetic—that govern the onset of instabilities at the interface. Finally, a comprehensive comparative study of SEI failure in Na and lithium (Li) metal anodes illustrates that the electrochemical and mechanical characteristics of the SEI are crucial in tailoring the anode morphology and interface stability. This work delineates mechanistic stability regimes cognizant of the SEI attributes and underlying failure modes and offers important guidelines for the design of artificial SEI layers for stable Na metal electrodes.

## Graphical abstract

The electrochemical and structural heterogeneities in the solid electrolyte interphase (SEI) influence the reaction kinetics of Na metal electrodes, resulting in current focusing and stress hotspots during electrodeposition. This work delineates mechanistic stability regimes cognizant of the SEI attributes and underlying failure modes and offers important guidelines for the design of artificial SEI layers for stable Na metal electrodes.

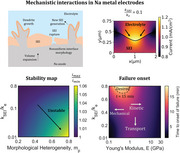

## 1 Introduction

The pursuit of sustainable energy technologies has increased the demand for efficient, cost‐effective, and eco‐friendly energy storage solutions.^[^ ^1^, ^2^, ^3^ ^]^ While Li‐ion batteries (LIBs) have garnered tremendous success in the past three decades, they encounter key hurdles such as the adverse environmental impacts of Li,^[^ ^4^, ^5^, ^6^ ^]^ high cost,^[^ ^7^, ^8^ ^]^ and safety issues.^[^ ^9^, ^10^, ^11^, ^12^, ^13^, ^14^, ^15^, ^16^ ^]^ In this context, Na‐based batteries have emerged as a compelling candidate due to the low cost and high abundance of Na.^[^ ^17^, ^18^, ^19^, ^20^ ^]^ In particular, the Na metal anode seems a promising anode material owing to its high theoretical specific energy (1166 mAh g^−1^) and a low electrochemical potential (−2.714 V vs standard hydrogen electrode).^[^ ^21^ ^]^ The Na metal anode has been found to show better performance with ether‐based electrolytes compared to other compatible anode materials favoring Na intercalation, such as hard carbon, metal oxides and sulfides, and alloys.^[^ ^22^, ^23^, ^24^, ^25^ ^]^ Recent efforts have also aimed at enabling Na–sulfur, Na–air batteries, and anode‐free Na metal batteries to further increase the energy density and reduce cost.^[^ ^26^, ^27^, ^28^ ^]^ Therefore, Na metal batteries (SMBs) offer an alluring alternative to conventional LIBs due to their high abundance, low cost and enhanced safety.

Despite these advantages, Na metal batteries exhibit various mechanistic challenges. For instance, Na readily reacts with electrolyte to form an irreversible and heterogeneous SEI at the anode.^[^ ^29^, ^30^, ^31^, ^32^ ^]^ The composition and structure of the SEI affect the plating/stripping mechanisms and govern the morphological evolution of the Na electrode.^[^ ^31^, ^33^ ^]^ An ideal SEI must be mechanically stable, ionically conductive, and electronically insulating, and thus mitigate dendrite growth, short‐circuits, and cell degradation. The electrolyte‐electrode pair plays a major role in determining the structure, composition, and properties of SEI.^[^ ^31^, ^34^ ^]^ The electrolyte solvation structure also affects the reduction potential of electrolyte, resulting in different SEI components.^[^ ^35^, ^36^, ^37^, ^38^ ^]^ Various research efforts have focused on optimizing the chemical composition of the SEI via electrolyte and interfacial modification strategies. For example, ether‐based electrolytes can form more stable passivating layers on the anode interface compared to carbonate‐based electrolytes.^[^ ^32^, ^39^ ^]^ It has also been reported that modulation of the electrolyte by adding fluorinated and polysulfide additives aids in stabilizing the SEI.^[^ ^40^, ^41^, ^42^ ^]^ In addition, sodiophilic artificial layers and surface coatings have been observed to promote stable deposition and suppress dendrite growth.^[^ ^43^, ^44^, ^45^, ^46^, ^47^ ^]^

The large volume changes of the Na anode during plating/stripping can induce cracks in SEI and lead to cell failure.^[^ ^48^, ^49^, ^50^, ^51^ ^]^ The SEI rupture results in exacerbated dendrite growth at the exposed Na metal region. Previous studies have investigated the role of SEI properties and morphology on stress generation and propensity for failure in Li metal batteries.^[^ ^52^, ^53^, ^54^, ^55^, ^56^, ^57^ ^]^ However, due to the distinct electrochemical/mechanical properties and composition of the SEI and the higher volume changes of Na, the underlying kinetic‐transport‐mechanics interactions and failure mechanisms in Na metal cells can be significantly different than Li metal cells. Furthermore, the Sand's time model for predicting dendrite growth may not be directly applicable to such Na metal systems due to the complex nature of heterogeneities in SEI that govern the interface morphology.^[^ ^32^ ^]^ Therefore, a detailed understanding of how different SEI characteristics dictate the coupled electro‐chemo‐mechanical interactions at the anode–electrolyte interface is required. In this work, we provide comprehensive insights into the influence of electrochemical and morphological heterogeneities in the SEI on the Na electrodeposition behavior and resulting interface instabilities. The spatial variations in the SEI promote more current focusing in low‐resistance SEI regions and the induced reaction heterogeneity plays a critical role in uneven interface growth. Notably, we reveal that the nonuniform spatiotemporal evolution of the mechanical stresses in SEI and Na metal strongly influences the mechanical overpotential, further amplifying the reaction heterogeneity and leading to faster filament growth. We also delineate the role of morphology and mechanical/transport properties of the SEI, such as ionic conductivity and Young's modulus, in governing different regimes of interface growth and failure onset. Finally, a thorough comparative analysis is performed to investigate the onset of failure in Na metal and Li metal anodes, revealing three distinct failure modes of the SEI: mechanical, transport, and kinetic.

## 2 Experimental Section

**Figure 1:**
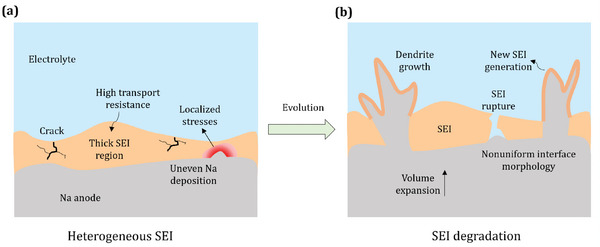
Schematic illustration of the a) electro‐chemo‐mechanical interactions at the Na metal/electrolyte interface for a nonuniform SEI and b) resulting failure mechanisms.

**Figure** **1**a shows a schematic illustration of the Na metal anode, electrolyte, and SEI formed at the interface. The SEI is considered to be ionically conductive but electronically insulating and different components in the SEI can give rise to spatially varying electrochemical and mechanical properties such as ionic conductivity and Young's modulus.

The diffusion of Na ions in the electrolyte and SEI was modeled using the Nernst–Plank equation based on mass conservation of chemical species:

(1) $$\frac{\partial c_{i}}{\partial t}+\nabla .\left(D_{i}\;\nabla c_{i}\right)=0$$

where *c_i_* is the ionic concentration and *D_i_* is the mass diffusivity of the electrolyte and different components of SEI.

The ionic transport is governed by migration due to electric potential gradients and diffusion due to concentrations gradients. The charge conservation of Na ions in the electrolyte and SEI is governed by:

(2) $$\nabla .\left(\kappa_{i}\;\nabla \phi_{i}\right)+\nabla .\left(\kappa_{D,i}\nabla \ln c_{i}\right)=0$$

where κ_*i*_ and κ_*D*, *i*_ are the ionic and diffusional conductivity, ϕ_*i*_ is the electric potential, and *c_i_* is the concentration of the electrolyte and different components of SEI.

The SEI typically has a low ionic conductivity, and different components of the SEI can also show a difference of up to three orders of magnitude in properties such as diffusivity and conductivity.^[^ ^58^, ^59^, ^60^, ^61^ ^]^ The ratio of ionic conductivity of the SEI and electrolyte, *k_SEI_*/*k_Elec_*, was considered to be 0.1 in this study unless stated otherwise.

The irregular deposition at the Na/SEI interface can induce large mechanical stresses in the SEI and Na metal. The stress distribution was calculated by solving the force balance equation for a linear elastic isotropic medium:

(3) ∇.σ=0

(4) $$\sigma =\frac{2\nu \mu \;}{1-2v}tr\left(\varepsilon \right)+2\mu \varepsilon$$

where ***σ*** is the Cauchy stress tensor, ***ε*** is the strain tensor, μ is the shear modulus, and ν is the Poisson's ratio of SEI or Na. It is noted that at high stresses, both SEI and Na may exhibit a nonlinear mechanical behavior and as a result, may undergo viscoplastic deformation over time. Moreover, stresses at the interface influence the reaction kinetics and, therefore, the interface growth can change with time depending on the nonlinear mechanical response. For this study, the SEI and Na were assumed to exhibit linear, elastic behavior.

Typically, the electrochemical potential is defined in terms of concentration and electric potential. However, stresses induced due to the mechanical deformation alter the energy landscape, impacting the kinetics of charge transfer reactions.^[^ ^62^, ^63^, ^64^ ^]^ The mechanical overpotential accounts for this change in energy due to stress variation and selectively contributes to the forward reaction.^[^ ^62^, ^65^ ^]^ The reaction kinetics at the Na metal interface is then given by the modified Butler–Volmer equation with contributions from both electric and mechanical overpotentials:^[^ ^66^ ^]^

(5) $$i_{rxn}=i_{0}\left\{exp\left(\frac{F\eta_{\phi }}{2RT}\right)exp\left(\frac{F\eta_{\sigma }}{RT}\right)-exp\left(-\frac{F\eta_{\phi }}{2RT}\right)\right\}$$

where *i_rxn_* is the reaction current density, *i*_0_ is the exchange current density, η_ϕ_ is the electric overpotential, η_σ_ is the mechanical overpotential, 𝐹 is the Faraday's constant, 𝑅 is the universal gas constant, and 𝑇 is the temperature. The electric and mechanical overpotentials at the anode interface are defined as:

(6) $$\eta_{\phi }=\phi_{s}-\phi_{e}-U$$

(7) $$\eta_{\sigma }=\frac{\Omega_{Na^{+}}\sigma_{h,e}-\Omega_{Na}\sigma_{h,Na}}{F}$$

where ϕ_*s*_ is the electric potential in metal anode, ϕ_*e*_ is the electric potential in SEI, *U* is the equilibrium potential, $\;\Omega_{Na^{+}}$ and Ω_*Na*_ are the partial molar volumes of Na^+^ in SEI and Na anode near the interface, respectively, and σ_*h*,*e*_ and σ_*h*,*Na*_ are the hydrostatic stresses in the SEI and Na anode, respectively. At high overpotentials, the reaction and transport heterogeneity will be higher due to the formation of different interphases and side products. To capture these intricacies, the spatiotemporal heterogeneities in electrochemical transport and mechanical properties, such as exchange current density and molar volume, should be considered.

The morphological evolution of the Na anode is governed by the electrodeposition of Na over time:

(8) $$\frac{\partial h}{\partial t}=\frac{-\;i_{rxn}\;\Omega_{Na}\;}{zF}$$

where *h* is the height of deposited Na, *t* is the time, and *z* is the number of valence electrons.

A detailed description of the parameters used in the modeling framework has been provided in Section S1 and Table S1 (Supporting Information). The associated boundary conditions used in the model are presented in Section S2 (Supporting Information).

## 3 Results and Discussion

Figure 1 describes the mechanistic challenges pertaining to the stability of the SEI at Na metal anode interface. For an ionically conductive SEI with perfectly uniform and favorable electrochemical, mechanical, and transport attributes, a stable interface evolution can be achieved during the electrodeposition process. However, an actual SEI, owing to its nonuniform morphology and spatially varying composition, can give rise to highly heterogeneous electro‐chemo‐mechanical interactions at the Na metal/electrolyte interface, resulting in the unstable interface evolution (Figure 1a). The low conductivity of the SEI results in sluggish transport in thicker SEI regions. The subsequent nonuniform nature of electrodeposition can make the interface more irregular and thus induce stress hotspots in the SEI. The fracture toughness of the SEI determines the critical stress limit beyond which onset of failure occurs.^[^ ^67^ ^]^ Cracks developed due to locally high mechanical stresses can propagate through the SEI ultimately leading to its rupture (Figure 1b). Subsequently, exposure of fresh Na to the electrolyte can trigger failure modes such as dendrite growth and short circuit. Additionally, the new SEI growth in these regions can result in lower coulombic efficiency, which can further exacerbate with repeated electrochemical cycling. In the subsequent sections, the effects of the morphological, electrochemical, and mechanical heterogeneities of the SEI on the interface stability have been investigated in detail.

**Figure 2:**
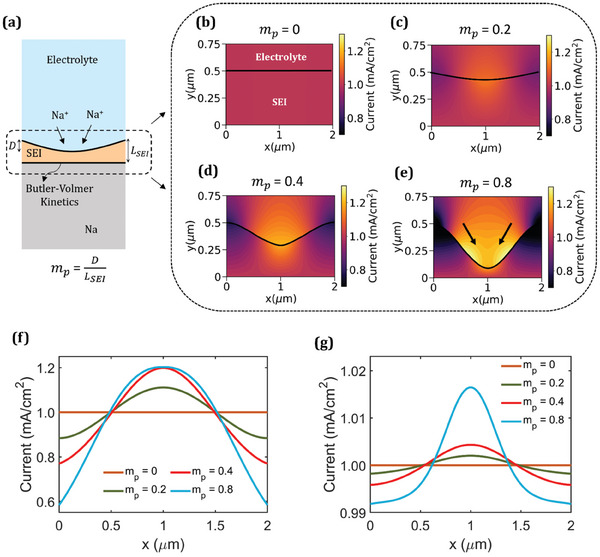
a) Computational modeling domain consisting of the SEI, electrolyte, and Na metal. Current distribution in SEI and electrolyte for different values of morphology parameter, *m*_*p*_ *= D*/*L_SEI_*: b) *m*_*p*_ *=* 0, c) *m*_*p*_ *=* 0.2, d) *m*_*p*_ *=* 0.4, and e) *m*_*p*_ *=* 0.8. f) Current distribution at SEI/electrolyte interface and g) reaction current density at the Na/SEI interface for different values of *m_p_*.

The modeling domain considered to investigate the role of SEI in Na metal anode stability is depicted in **Figure** **2**a. The morphological heterogeneity is given by the morphology parameter, *m*_*p*_ *= D*/*L_SEI_*, where *D* is the difference between the maximum and minimum SEI thickness, and *L_SEI_* is the maximum SEI thickness. Figure 2b–e shows the current distribution profiles within the SEI and electrolyte for morphology parameters, *m*_*p*_ *=* 0, 0.2, 0.4, and 0.8 for an applied current density, *I_App_* = 1 mA cm^−2^. The locally thicker SEI regions have a higher ion transport resistance and therefore a nonuniform SEI morphology leads to a higher current focusing in regions of lesser SEI thickness.

The current distribution at the SEI/electrolyte interface for different morphology parameters is shown in Figure 2f. For a higher morphological heterogeneity, the extreme values of current density deviate significantly from the applied current density (1 mA cm^−2^). The maximum and minimum values of current density at the SEI/electrolyte interface reach 1.2 and 0.6 mA cm^−2^, respectively, for *m*_*p*_ *=* 0.8. Subsequently, the heterogeneity in the current distribution within the SEI propagates to the Na/SEI interface and manifests into a nonuniform electrodeposition pattern, which is evident from the reaction distribution shown in Figure 2g. The maximum current density at the Na/SEI interface is 1.017 mA cm^−2^ for *m*_*p*_ *=* 0.8. While this is not as high as the maximum current density at the SEI/electrolyte interface, it is sufficient to trigger an instability over time due to SEI fracture and dendrite growth as discussed later. Figure S1a–d (Supporting Information) shows the electric potential distribution within the SEI and the electrolyte for different morphology parameters. The potential drop at the center is higher for a more nonuniform SEI morphology due to current focusing in thinner regions. The electric overpotential at the Na/SEI interface is also higher in these regions (Figure S2a, Supporting Information), resulting in a higher interfacial resistance. Overall, the results presented in Figure 2 indicate that the SEI morphology plays a critical role in dictating the stability of the Na metal anode.

**Figure 3:**
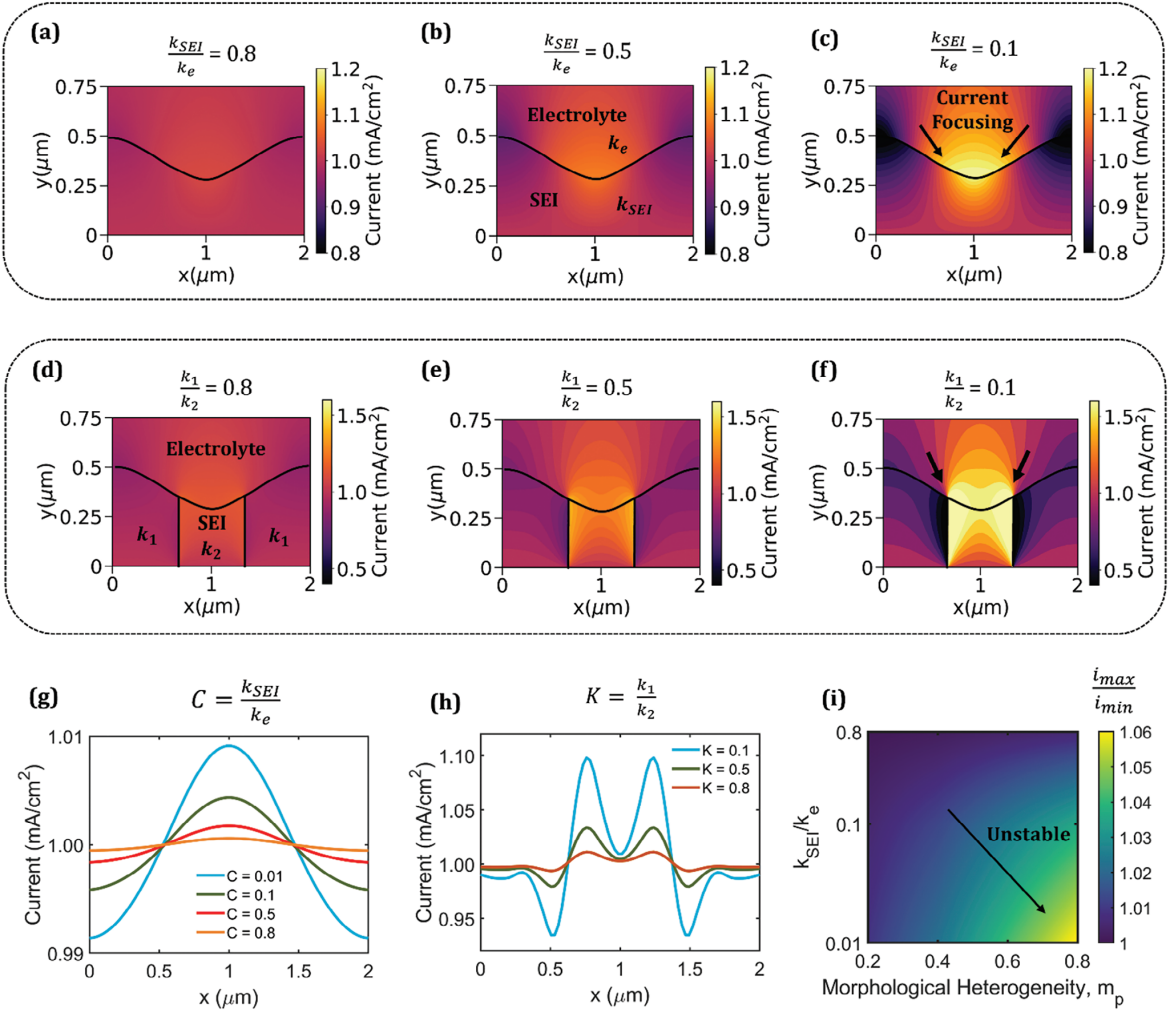
Current distribution for different SEI ionic conductivities (denoted by *C = k_SEI_*/*k_elec_*): a) *C =* 0.8, b) *C =* 0.5, and c) *C =* 0.1. Current distribution for different values of spatial heterogeneity in SEI conductivity (denoted by *K*  =  *k*_1_/*k*_2_): d) *K*  =  0.8, e) *K*  =  0.5, and f) *K*  =  0.1. Reaction current density at the Na/SEI interface for different values of g) SEI ionic conductivity and h) SEI spatial heterogeneity. i) Effect of ionic conductivity and morphology of SEI on the stability parameter, θ  = *i_max_* /*i_min_*. All plots for *I_App_* = 1 mA cm^−2^, *m_p_* = 0.4.

In addition to the SEI morphology, we examine the underpinning role of the ionic conductivity of the SEI on the electrodeposition behavior of Na. The ionic conductivity of the SEI is expressed as the nondimensional parameter, *C = k_SEI_*/*k_Elec_*, where *k_SEI_* and *k_Elec_* are the ionic conductivities of the SEI and electrolyte, respectively. **Figure** **3**a–c shows the current profiles in the SEI and the electrolyte for three different values of SEI conductivity. A decrease in SEI conductivity promotes more current focusing toward thinner SEI regions and the maximum current density at the SEI/electrolyte interface exceeds 1.2 mA cm^−2^ as *C* decreases to 0.1. A more nonuniform overpotential distribution at the Na/SEI interface is also observed for a lower SEI conductivity (Figure S2b, Supporting Information). Furthermore, the presence of different SEI components can give rise to a spatial variation in ionic conductivity within the SEI. To investigate this effect, we consider three regions in the SEI with different ionic conductivities. *K = k*_1_/*k*_2_ is illustrative of the spatial heterogeneity, where *k*_1_ and *k*_2_ are the minimum and maximum ionic conductivities of SEI components, respectively. Figure 3d–f shows the current distribution for different magnitudes of spatial heterogeneity in the SEI. A strong correlation is observed between the spatial heterogeneity and the current distribution; specifically, regions of higher ionic conductivity give rise to high current densities at the SEI/electrolyte interface. For *K* = 0.1, the maximum current density at the interface exceeds 1.5 mA cm^−2^ whereas the minimum current density falls below 0.5 mA cm^−2^.

The transport nonuniformity stemming from a low SEI conductivity or high spatial heterogeneity propagates to the Na/SEI interface and alters the reaction current density (Figure 3g,h). However, the reaction distribution is different for the two scenarios. For a low SEI conductivity, higher current focusing is attained near the central low resistance region whereas when a spatial heterogeneity is present, the focusing is more pronounced near the interface of different SEI components due to sudden change in ionic conductivity. The current focusing is higher in these regions compared to regions of minimum thickness and results in peaks at the interface of different components. Moreover, it is observed that spatial variations in the SEI have a significantly higher effect on the reaction heterogeneity, whose magnitudes reach 1.1 mA cm^−2^ at the Na/SEI interface for *K =* 0.1 (Figure 3h).

The variation of the stability parameter, θ  =  *i_max_*/*i_min_*, with morphology and SEI ionic conductivity is shown in Figure 3i. The stability parameter denotes the ratio of maximum and minimum reaction current density at the Na/SEI interface. It is an indicator of the reaction and a value closer to 1 implies more uniform growth. The electrodeposition instability increases for a lower SEI ionic conductivity and a higher morphological heterogeneity. A significant increase in instability (θ >  1.05) is attained for morphology parameters (*m_p_*) beyond 0.6, especially for low values of SEI ionic conductivity (*C =* 0.01). However, relatively stable electrodeposition is achieved at a low *m_p_* (<0.4) even for a low SEI ionic conductivity. Therefore, both electrochemical and mechanical properties together govern the interface stability, and a uniform SEI morphology promotes more homogenous reaction distribution at the Na/SEI interface.

**Figure 4:**
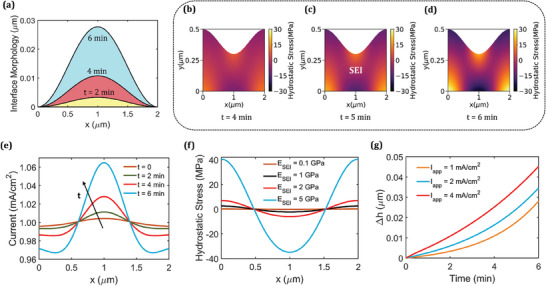
a) Na/SEI interface morphology, b–d) hydrostatic stress distribution, and e) reaction current density at the Na/SEI interface at different times, *E_SEI_* = 2 GPa. f) Hydrostatic stress distribution at the interface for different values of Young's modulus (*E_SEI_*) at time, *t* = 3 min. g) Evolution of protrusion height with time for different applied current densities. All plots for *k_SEI_*/*k_elec_* = 0.1, *m_p_* = 0.4.

The nonuniform reaction distribution will lead to a heterogeneous interface growth at the Na anode interface. **Figure** **4**a and Figure S3 (Supporting Information) shows the dynamic evolution of the Na/SEI interface during the electrodeposition process for *I_App_* = 1 mA cm^−2^, *m_p_* = 0.4, *k_SEI_*/*k_elec_* = 0.1, and *E_SEI_* = 2 GPa, where *E_SEI_* denotes the Young's modulus of the SEI. We observe that the induced reaction heterogeneity results in a nonuniform Na deposition and protrusion growth. Subsequently, the uneven volume change of Na due to preferential electrodeposition gives rise to localized stresses in SEI and Na. Even though there is no applied pressure, the nonuniform deposition and localized deformations generate a heterogeneous stress distribution in the SEI. Figure 4b–d shows the hydrostatic stress distribution within the SEI at times, *t* = 4, 5, and 6 min. At the Na/SEI interface, compressive stresses build up at the locations with higher Na deposition (i.e., center of the interface). On the other hand, tensile stresses develop at the locations with lower Na deposition (i.e., edges of the Na/SEI interface) as observed in Figure 4b–d. The magnitudes of both maximum compressive and tensile stresses exceed 30 MPa at *t* = 6 min. It is important to note that significant induced stresses due to mechanical deformation alter the energy landscape, impacting the kinetics of charge transfer reactions. The mechanical overpotential accounts for the effect of stresses at the interface on reaction kinetics and thereby influences the electrodeposition behavior. In particular, preferential deposition takes places in regions of higher compressive stresses in the SEI due to more negative mechanical overpotentials in these areas which increases the forward rate reaction (see Equation 5). In regions of tensile stresses, the mechanical overpotential is positive and therefore results in decreased forward reactions rates and lesser deposition. Therefore, the induced mechanical stresses direct Na deposition in regions of lesser SEI thickness, further exacerbating the interface instability. Figure 4e illustrates this evolution of reaction current density at times, *t* = 0, 2, 4, and 6 min. The increase in reaction heterogeneity from *t* = 4 to 6 min is almost twice as much as the increase in heterogeneity from *t* = 2 to 4 min. In the present study, only deposition behavior of sodium metal at the anode during charging has been analyzed. During dissolution, the stress distribution at the interface will differ due to distinct morphological evolution. The regions of compressive and tensile stresses will vary, thereby altering the reaction current density and growth rate.

To further examine the effect of mechanical overpotential on the interface stability, Figure 4f shows the hydrostatic stress distribution for different values of *E_SEI_* at time, *t* = 3 min. The induced stresses are higher for a greater SEI modulus and reach up to 40 MPa for *E_SEI_* = 5 GPa. This results in an increase in the mechanical overpotential and subsequently more nonuniformity in the electrodeposition. Therefore, the heterogeneous interface growth is faster for a higher *E_SEI_* (Figure S4a, Supporting Information). However, a higher *E_SEI_* can also help in preventing material failure due to enhanced strength and the overall influence of *E_SEI_* on onset of failure is discussed ahead. Lastly, the effect of applied current density (*I_App_*) on morphological evolution (given by Δ*h*) is shown in Figure 4g for *I_App_* = 1, 2, and 4 mA cm^−2^. Here, Δ*h* denotes the protrusion height of deposited Na at the interface. The protrusion growth exceeds 0.04 µm within 6 min for *I_App_* = 4 mA cm^−2^ which will result in high localized stresses and early failure of SEI.

**Figure 5:**
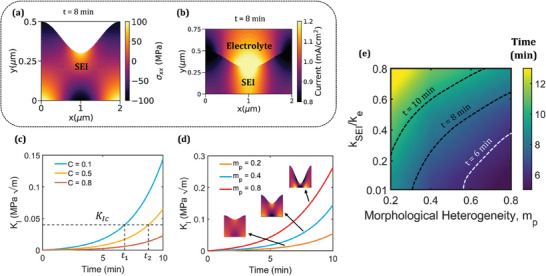
a) Normal stress along X‐axis and b) current distribution in the SEI at time, *t* = 8 min. Evolution of Mode I stress intensity for different values of c) ionic conductivity of SEI (denoted by *C = k_SEI_*/*k_elec_*) and d) morphological heterogeneity. e) Effect of ionic conductivity and morphology of SEI on time to onset of failure.

As the interface becomes more heterogeneous over time, the high tensile normal stresses along X‐axis (σ_*xx*_) can initiate cracks in the SEI. The normal stress and current distribution in the SEI at time, *t* = 8 min are shown in **Figure** **5**a,b. It can be seen that the magnitudes of maximum compressive and tensile normal stresses in the SEI exceed 100 MPa (Figure 5a). The high stresses alter the mechanical overpotential, resulting in a highly heterogeneous reaction current density at the anode interface (Figure 5b). The onset of failure is determined by modified form of Griffith's criterion based on Mode I stress intensity (*K_I_*). The value of *K_I_* depends on strain energy release rate (*G*) and Young's modulus (*E*) and is given by $K_{I}=\sqrt{G.E/(1-\nu^{2}})$, where ν is the Poisson's ratio. The strain energy release rate is calculated using the J‐integral based on stresses and deformation around the protrusion.^[^ ^68^ ^]^ The crack initiation occurs when *K_I_* exceeds the fracture toughness (*K_Ic_*), which is a measure of strength of the material (details in Section S3, Supporting Information).

Figure S5 (Supporting Information) shows the maximum hydrostatic stress evolution in the SEI whereas Figure 5c,d shows the evolution of *K_I_* for different values of SEI ionic conductivity and morphology parameters, *m_p_*. Since a lower ionic conductivity of SEI and higher morphological heterogeneity promote faster growth of the instability, the time to reach the critical stress intensity (*K_Ic_*) is lesser for a low *k_SEI_* and high *m*_*p*_ (e.g., 8.8 min for *k_SEI_*/*k_Elec_* = 0.5 vs 6.8 min for *k_SEI_*/*k_Elec_* = 0.1 at *m*_*p*_= 0.4). Figure 5e shows the combined effect of *m*_*p*_and *I_App_* on the time to onset of failure. The failure onset can occur within 5 min at a high *m*_*p*_(> 0.6) for *k_SEI_*/*k_Elec_* = 0.1 due to severe current focusing and high stresses. Altogether, the structural and mechanical characteristics of the SEI can amplify heterogeneities over time and trigger the onset of failure which will further result in SEI rupture and dendrite growth.

**Figure 6:**
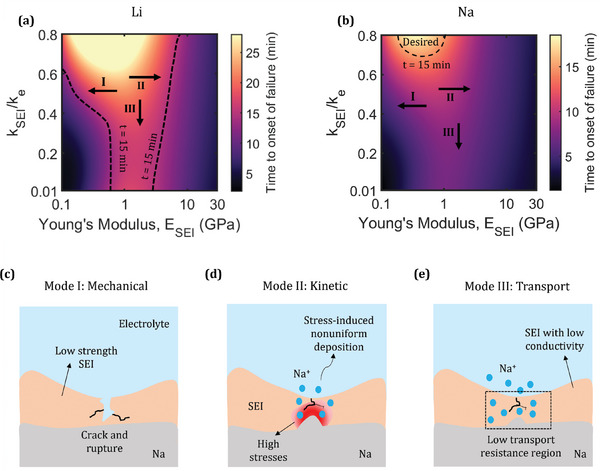
Time to onset of failure for different values of ionic conductivity and Young's modulus of the SEI for a) Li and b) Na. Different failure modes: c) mechanical, d) kinetic, and e) transport.

Finally, we present a detailed comparison between Na and Li metal anodes. The mechanics, reaction kinetics, and transport are altered by the metal properties, such as Young's modulus and molar volume. This is evident in Figure S6 (Supporting Information) which shows that Na undergoes a faster interface growth than Li. **Figure** **6**a,b shows the time to onset of failure (*t_f_*) for different values of ionic conductivity (*k_SEI_*/*k_elec_*) and Young's modulus of SEI (*E_SEI_*) for Li and Na metal anodes at *I_App_* = 1 mA cm^−2^, *m_p_* = 0.4. We identify three different modes governing onset of SEI failure: mechanical, transport, and kinetic. For low values of *E_SEI_*, the SEI is not able to sustain high stresses due to its low mechanical strength (Section S3, Supporting Information). Therefore, crack initiation occurs even for low stresses in the SEI which further results in SEI rupture. The onset time is <5 min for *E_SEI_* = 0.1 GPa for both Li and Na (Figure 6c). On the other hand, for high values of *E_SEI_*, the generated stresses and mechanical overpotentials are very high (Figure 4f). This kinetic heterogeneity results in a nonuniform reaction current density and uneven electrodeposition at the interface (Figure 6d). The strength of the SEI also increases with *E_SEI_* but it is not sufficient to counter the mechanical overpotential effects and hence a decrease in *t_f_* is observed with a further increase in *E_SEI_* (Figure S4b, Supporting Information). For *E_SEI_* > 10 GPa, *t_f_* drops below 5 min even for a high SEI conductivity. Therefore, the maximum stability (high *t_f_*) is obtained for moderate values of *E_SEI_* ≈1 GPa. This value shifts slightly to the left for Na (≈0.5 GPa) because high stresses develop rapidly even for a lower *E_SEI_* due to faster protrusion growth at the Na interface.

A decrease in ionic conductivity of the SEI induces a higher reaction nonuniformity (Figure 3g) and reduced onset time (Figure 5c). Thus, the nonuniform transport in the SEI exacerbates for low SEI ionic conductivity (Figure 6e) and *t_f_* reaches below 3 min for *k_SEI_*/*k_elec_* <0.1 at extremely high or low values of *E_SEI_*. Therefore, an optimal regime for Na with a high *t_f_* is obtained for a moderate *E_SEI_* of around 0.5 GPa and *k_SEI_*/*k_elec_* > 0.7. For Li, the preferred regime is much larger due to slower filament growth. Beyond *t_f_*, cracks will propagate through the SEI and rupture it. The high current density at the ruptured region will induce dendrite growth and the reaction of metal with the electrolyte at the exposed region will result in regeneration of SEI. This cycle of heterogeneous growth, rupture and new SEI generation will eventually result in cell failure.

An actual SEI is highly heterogeneous, characterized by surface roughness, grain boundaries, and variability in composition. The heterogeneities can trigger instabilities, resulting in uneven growth and failures at specific regions. These localized effects can be understood by the mechanisms established in this study. The trends observed in this study for different types of heterogeneities are also broadly applicable. In our analysis, we provide a range of the structural and electrochemical properties of SEI that can aid in analyzing different systems. However, based on the underlying transport–kinetic–mechanics coupling, the regimes of interface stability may change. For example, in intercalation‐based electrode materials (e.g., graphite, NMC), diffusion‐induced stress and its effect on the reaction kinetics and transport mechanisms need to be considered. Whereas in high‐capacity anode materials (e.g., Sn), large volume expansion and its impact on SEI instabilities need to be considered. While the modeling framework presented in this study is applicable to the metal electrode systems, by incorporating the mechanisms specific to another electrode system, the analysis can be extended to study the interfacial stability phenomenon in other electrode materials.

## 4 Conclusion

This work presents a coupled electro‐chemo‐mechanical modeling framework to investigate the underlying role of SEI heterogeneities on the morphological evolution of the Na metal/electrolyte interface. We demonstrate that heterogeneities in the SEI induce a reaction heterogeneity at the interface due to current focusing in SEI regions with less resistance. The electrodeposition instability critically increases for a lower SEI ionic conductivity and a greater spatial variation in SEI properties. Through our analysis, we reveal that the buildup of higher mechanical stresses and mechanical overpotentials at the Na/SEI interface is a pivotal factor that results in nonuniform interface morphology and an increase in the reaction heterogeneity over time. Depending on the electrochemical and mechanical characteristics of the SEI, three distinct failure modes can arise, namely, mechanical, kinetic, and transport. A low modulus SEI fails early due to its low mechanical strength whereas a high modulus SEI undergoes faster filament growth due to higher stresses and enhanced kinetic heterogeneity at the interface. Na undergoes a faster filament growth compared to Li and requires a more conductive, uniform SEI for stable interface growth. More specifically, a moderate SEI Young's modulus, *E_SEI_* of ≈0.5 GPa and an ionic conductivity of SEI, *k_SEI_*/*k_elec_* > 0.7 can tailor stable Na electrodeposition. The demarcation of such optimal growth regimes offers crucial guidelines for the design of artificial SEI layers to enable stable Na metal electrodes.

## Conflict of Interest

The authors declare no conflict of interest.

## Supporting information

Supporting Information

## Data Availability

The data that support the findings of this study are available from the corresponding author upon reasonable request.

## References

[1] C. P. Grey , D. S. Hall , Nat. Commun. 2020, 11, 6279. 33293543 10.1038/s41467-020-19991-4 PMC7722877

[2] O. Schmidt , S. Melchior , A. Hawkes , I. Staffell , Joule 2019, 3, 81.

[3] Y. Tian , G. Zeng , A. Rutt , T. Shi , H. Kim , J. Wang , J. Koettgen , Y. Sun , B. Ouyang , T. Chen , Z. Lun , Z. Rong , K. Persson , G. Ceder , Chem. Rev. 2021, 121, 1623. 33356176 10.1021/acs.chemrev.0c00767

[4] D. B. Agusdinata , W. Liu , H. Eakin , H. Romero , Environ. Res. Lett. 2018, 13, 123001.

[5] V. Flexer , C. F. Baspineiro , C. I. Galli , Sci. Total Environ. 2018, 639, 1188. 29929287 10.1016/j.scitotenv.2018.05.223

[6] R. B. Kaunda , J. Energy Nat. Resour. Law 2020, 38, 237.

[7] J.‐M. Tarascon , Nat. Chem. 2010, 2, 510. 20489722 10.1038/nchem.680

[8] R. Schmuch , R. Wagner , G. Hörpel , T. Placke , M. Winter , Nat. Energy 2018, 3, 267.

[9] R. Srinivasan , P. A. Demirev , B. G. Carkhuff , S. Santhanagopalan , J. A. Jeevarajan , T. P. Barrera , J. Electrochem. Soc. 2020, 167, 140516.

[10] J. Zhang , L. Zhang , F. Sun , Z. Wang , IEEE Access 2018, 6, 23848.

[11] J. B. Goodenough , Y. Kim , Chem. Mater. 2009, 22, 587.

[12] B. S. Vishnugopi , F. Hao , A. Verma , P. P. Mukherjee , ACS Appl. Mater. Interfaces 2020, 12, 23931. 32363849 10.1021/acsami.0c04355

[13] A. Patil , V. Patil , D. Wook Shin , J.‐W. Choi , D.‐S. Paik , S.‐J. Yoon , Mater. Res. Bull. 2008, 43, 1913.

[14] J. M. Tarascon , M. Armand , Nature 2001, 414, 359. 11713543 10.1038/35104644

[15] B. S. Vishnugopi , E. Kazyak , J. A. Lewis , J. Nanda , M. T. McDowell , N. P. Dasgupta , P. P. Mukherjee , ACS Energy Lett. 2021, 6, 3734.

[16] J. Wen , Y. Yu , C. Chen , Mater. Express 2012, 2, 197.

[17] D. Larcher , J. M. Tarascon , Nat. Chem. 2015, 7, 19. 25515886 10.1038/nchem.2085

[18] H. Pan , Y.‐S. Hu , L. Chen , Energy Environ. Sci. 2013, 6, 2338.

[19] C. Vaalma , D. Buchholz , M. Weil , S. Passerini , Nat. Rev. Mater. 2018, 3, 18013.

[20] V. Palomares , M. Casas‐Cabanas , E. Castillo‐Martínez , M. H. Han , T. Rojo , Energy Environ. Sci. 2013, 6, 2312.

[21] N. Yabuuchi , K. Kubota , M. Dahbi , S. Komaba , Chem. Rev. 2014, 114, 11636. 25390643 10.1021/cr500192f

[22] Z. W. Seh , J. Sun , Y. Sun , Y. Cui , ACS Cent. Sci. 2015, 1, 449. 27163006 10.1021/acscentsci.5b00328 PMC4827673

[23] B. Sun , P. Xiong , U. Maitra , D. Langsdorf , K. Yan , C. Wang , J. Janek , D. Schroder , G. Wang , Adv. Mater. 2020, 32, 1903891. 10.1002/adma.201903891 31599999

[24] Y. Fang , L. Xiao , Z. Chen , X. Ai , Y. Cao , H. Yang , Electrochem. Energy Rev. 2018, 1, 294.

[25] J. Y. Hwang , S. T. Myung , Y. K. Sun , Chem. Soc. Rev. 2017, 46, 3529. 28349134 10.1039/c6cs00776g

[26] P. Adelhelm , P. Hartmann , C. L. Bender , M. Busche , C. Eufinger , J. Janek , Beilstein J. Nanotechnol. 2015, 6, 1016. 25977873 10.3762/bjnano.6.105 PMC4419580

[27] A. P. Cohn , N. Muralidharan , R. Carter , K. Share , C. L. Pint , Nano Lett. 2017, 17, 1296. 28112523 10.1021/acs.nanolett.6b05174

[28] Z. Hu , L. Liu , X. Wang , Q. Zheng , C. Han , W. Li , Adv. Funct. Mater. 2024, 34, 2313823.

[29] E. Peled , S. Menkin , J. Electrochem. Soc. 2017, 164, A1703.

[30] L. Fan , X. Li , Nano Energy 2018, 53, 630.

[31] E. Matios , H. Wang , C. Wang , W. Li , Ind. Eng. Chem. Res. 2019, 58, 9758.

[32] B. Lee , E. Paek , D. Mitlin , S. W. Lee , Chem. Rev. 2019, 119, 5416. 30946573 10.1021/acs.chemrev.8b00642

[33] X. Zheng , C. Bommier , W. Luo , L. Jiang , Y. Hao , Y. Huang , Energy Storage Mater. 2019, 16, 6.

[34] G. G. Eshetu , T. Diemant , M. Hekmatfar , S. Grugeon , R. J. Behm , S. Laruelle , M. Armand , S. Passerini , Nano Energy 2019, 55, 327.

[35] T. Cai , Y. Wang , F. Zhao , Z. Ma , P. Kumar , H. Xie , C. Sun , J. Wang , Q. Li , Y. Guo , J. Ming , Adv. Energy Mater. 2024, 14, 2400569.

[36] Z. Tian , Y. Zou , G. Liu , Y. Wang , J. Yin , J. Ming , H. N. Alshareef , Adv. Sci. (Weinh) 2022, 9, e2201207. 35661442 10.1002/advs.202201207 PMC9353483

[37] Q. Li , Z. Cao , H. Cheng , J. Zhang , Z. Ma , W. Wahyudi , L. Cavallo , Q. Sun , J. Ming , ACS Mater. Lett. 2022, 4, 2469.

[38] L. Zhou , Z. Cao , J. Zhang , Q. Sun , Y. Wu , W. Wahyudi , J. Y. Hwang , L. Wang , L. Cavallo , Y. K. Sun , H. N. Alshareef , J. Ming , Nano Lett. 2020, 20, 3247. 32319776 10.1021/acs.nanolett.9b05355

[39] C. Bao , B. Wang , P. Liu , H. Wu , Y. Zhou , D. Wang , H. Liu , S. Dou , Adv. Funct. Mater. 2020, 30, 2004891.

[40] H. Wang , C. Wang , E. Matios , W. Li , Angew. Chem. Int. Ed. Engl. 2018, 57, 7734. 29693763 10.1002/anie.201801818

[41] G. G. Eshetu , M. Martinez‐Ibanez , E. Sanchez‐Diez , I. Gracia , C. Li , L. M. Rodriguez‐Martinez , T. Rojo , H. Zhang , M. Armand , Chem. Asian J. 2018, 13, 2770. 30035860 10.1002/asia.201800839

[42] J. Fondard , E. Irisarri , C. Courrèges , M. R. Palacin , A. Ponrouch , R. Dedryvère , J. Electrochem. Soc. 2020, 167, 070526.

[43] L. Ye , M. Liao , T. Zhao , H. Sun , Y. Zhao , X. Sun , B. Wang , H. Peng , Angew. Chem. Int. Ed. Engl. 2019, 58, 17054. 31523899 10.1002/anie.201910202

[44] S. Tang , Z. Qiu , X.‐Y. Wang , Y. Gu , X.‐G. Zhang , W.‐W. Wang , J.‐W. Yan , M.‐S. Zheng , Q.‐F. Dong , B.‐W. Mao , Nano Energy 2018, 48, 101.

[45] J. Luo , C. Wang , H. Wang , X. Hu , E. Matios , X. Lu , W. Zhang , X. Tao , W. Li , Adv. Funct. Mater. 2019, 29, 1805946.

[46] B. Sun , P. Li , J. Zhang , D. Wang , P. Munroe , C. Wang , P. H. L. Notten , G. Wang , Adv. Mater. 2018, 30, 1801334. 10.1002/adma.201801334 29855109

[47] S. Wei , S. Choudhury , J. Xu , P. Nath , Z. Tu , L. A. Archer , Adv. Mater. 2017, 29, 1605512. 10.1002/adma.201605512 28112842

[48] J. Lee , J. Kim , S. Kim , C. Jo , J. Lee , Mater. Adv. 2020, 1, 3143.

[49] W. Liu , P. Liu , D. Mitlin , Adv. Energy Mater. 2020, 10, 2002297.

[50] M. Hou , Y. Zhou , F. Liang , H. Zhao , D. Ji , D. Zhang , L. Li , Y. Lei , Chem. Eng. J. 2023, 475, 146227.

[51] L. Ma , J. Cui , S. Yao , X. Liu , Y. Luo , X. Shen , J.‐K. Kim , Energy Storage Mater. 2020, 27, 522.

[52] X. Shen , R. Zhang , X. Chen , X. B. Cheng , X. Li , Q. Zhang , Adv. Energy Mater. 2020, 10, 1903645.

[53] Y. Liu , X. Xu , O. O. Kapitanova , P. V. Evdokimov , Z. Song , A. Matic , S. Xiong , Adv. Energy Mater. 2022, 12, 2103589.

[54] F. Hao , A. Verma , P. P. Mukherjee , J. Mater. Chem. A 2018, 6, 19664.

[55] K. G. Naik , B. S. Vishnugopi , J. Datta , D. Datta , P. P. Mukherjee , Appl. Mech. Rev. 2023, 75, 010802 .

[56] X. R. Chen , Y. X. Yao , C. Yan , R. Zhang , X. B. Cheng , Q. Zhang , Angew. Chem. Int. Ed. Engl. 2020, 59, 7743. 32160379 10.1002/anie.202000375

[57] B. Horstmann , J. Shi , R. Amine , M. Werres , X. He , H. Jia , F. Hausen , I. Cekic‐Laskovic , S. Wiemers‐Meyer , J. Lopez , D. Galvez‐Aranda , F. Baakes , D. Bresser , C.‐C. Su , Y. Xu , W. Xu , P. Jakes , R.‐A. Eichel , E. Figgemeier , U. Krewer , J. M. Seminario , P. B. Balbuena , C. Wang , S. Passerini , Y. Shao‐Horn , M. Winter , K. Amine , R. Kostecki , A. Latz , Energy Environ. Sci. 2021, 14, 5289.

[58] R. Guo , B. M. Gallant , Chem. Mater. 2020, 32, 5525.

[59] A. Ramasubramanian , V. Yurkiv , T. Foroozan , M. Ragone , R. Shahbazian‐Yassar , F. Mashayek , J. Phys. Chem. C 2019, 123, 10237.

[60] L. Benitez , J. M. Seminario , J. Electrochem. Soc. 2017, 164, E3159.

[61] H. Yildirim , A. Kinaci , M. K. Chan , J. P. Greeley , ACS Appl. Mater. Interfaces 2015, 7, 18985. 26255641 10.1021/acsami.5b02904

[62] C. Monroe , J. Newman , J. Electrochem. Soc. 2004, 151, A880.

[63] Y. Zhao , P. Stein , Y. Bai , M. Al‐Siraj , Y. Yang , B.‐X. Xu , J. Power Sources 2019, 413, 259.

[64] V. S. Deshpande , R. M. McMeeking , Appl. Mech. Rev. 2023, 75, 010801.

[65] M. Ganser , F. E. Hildebrand , M. Klinsmann , M. Hanauer , M. Kamlah , R. M. McMeeking , J. Electrochem. Soc. 2019, 166, H167.

[66] D. Chatterjee , K. G. Naik , B. S. Vishnugopi , P. P. Mukherjee , Adv. Sci. 2023, 11, e2307455. 10.1002/advs.202307455 PMC10853722 38072655

[67] K. Guo , R. Kumar , X. Xiao , B. W. Sheldon , H. Gao , Nano Energy 2020, 68, 104257.

[68] J. R. Rice , J. Appl. Mech. 1968, 35, 379.

