# Supplementary material for: Heterogeneous Solid Electrolyte Interphase Interactions Dictate Interface Instability in Sodium Metal Electrodes
Source: Adv Sci (Weinh). 2024 Jul 30;11(36):2404887. doi: 10.1002/advs.202404887 (PMC11423159; doi:10.1002/advs.202404887)
Supplement: Supplementary file 1 — Supporting Information [file ADVS-11-2404887-s001.docx]

**Supporting Information**

**Heterogeneous** **Solid Electrolyte Interphase Interactions Dictate Interface Instability in Sodium Metal Electrodes**

*Aditya Singla, Kaustubh G. Naik, Bairav S. Vishnugopi, and Partha P. Mukherjee^*^*

School of Mechanical Engineering, Purdue University, West Lafayette, IN 47907, USA

^*^*Correspondence*: pmukherjee@purdue.edu

**S1. Properties of solid electrolyte interphase (SEI)**

Table S1 lists the values of parameters used in this study. The SEI for Na-based systems is relatively thick consisting of multiple layers with varying composition and its thickness can range from tens of nanometers to up to a few micrometers. ^[1-4]^ In our model, the thickness of the SEI is assumed to be 500 nm and we study the effects of SEI in a region of width 2 $\mu m$ with electrolyte and sodium domain lengths as 10 $\mu m$. The electrochemical and mechanical properties of electrolyte and Na have been taken from studies on diglyme-based electrolytes and Na metal anode. ^[5-8]^ The properties of SEI correspond to the average properties of Na-based SEI components such as NaF, Na_2_O, Na_2_CO_3_ (taken from material property data).

**S2. Boundary and initial Conditions:**

The conditions for mass and charge transport have been incorporated from Barai et al. ^[9]^ A uniform current density and a fixed concentration gradient are applied at the top electrolyte boundary, and the Butler-Volmer kinetics is solved at the Na/SEI interface. At the left and right boundaries, no flux boundary condition has been imposed for both potential and concentration. The initial concentration is taken to be uniform throughout the electrolyte and the SEI.

The displacement along x-direction ($u_{x})$ is set to zero at the left and right boundaries and no pressure boundary condition has been applied at the top boundary of the electrolyte. The displacement along y-direction ($u_{y})$ is taken to be zero at the bottom Na boundary. At the Na/SEI interface, we equate the normal stresses along y-axis ($\sigma_{yy}$) in Na and SEI. The stresses in the liquid electrolyte are assumed to be negligible compared to stresses in Na and SEI.

**Table S1**. List of parameters and properties used in the model.

| **Parameters** |  | **Values** | **Units** |
| --- | --- | --- | --- |
| $L_{SEI}$ | Thickness of SEI | 500 | $nm$ |
| $w$ | Domain width | 2 | $\mu m$ |
| $L_{a}$ | Thickness of Na anode | 10 | $\mu m$ |
| $L_{e}$ | Thickness of electrolyte domain | 10 | $\mu m$ |
| $D_{e}$ | Diffusivity of electrolyte | 10^-10^ | m^2^ s^-1^ |
| $k_{e}$ | Ionic conductivity of electrolyte | 5 | mS cm^-1^ |
| $E_{Na}$ | Young’s modulus of Na | 4 | $\mathrm{GPa}$ |
| $\nu_{Na}$ | Poisson’s ratio of Na | 0.29 | $-$ |
| $\nu_{SEI}$ | Poisson’s ratio of SEI | 0.3 | $-$ |
| $\Omega_{Na}$ | Partial molar volume of Na | 23 | cm^3^ mol^-1^ |
| $\Omega_{{Na}^{+}}$ | Partial molar volume of $\mathrm{Na}^{+}$ ion in SEI | 16 | cm^3^ mol^-1^ |
| $i_{0}$ | Exchange current density | 1 | mA cm^-2^ |
| $c_{o}$ | Initial electrolyte and SEI concentration | 1000 | mol m^-3^ |
| $t_{f}$ | Charge Transfer Coefficient | 0.3 | - |
| $F$ | Faraday constant | 96,485 | C mol^-1^ |
| $T$ | Temperature | 298 | K |
| $R$ | Universal Gas Constant | 8.314 | J mol^-1^ K^-1^ |

**S3. Correlation between fracture toughness and Young’s modulus of SEI**

The strength of a material is given by its fracture toughness ($K_{Ic}$) which depends on the type of defect and failure. The correlation between $K_{Ic}$ and Young’s modulus ($E)$ is usually not simple and depends on various factors including the type of material. An approximate dependence can be described as $K_{Ic}= (b{E)}^{n}$, where $b$ and n are constants, $K_{Ic}$ is measured in MPa m^1/2^ and $E$ in GPa. ^[10-12]^ For this study, we assume $b=0.1$ and $n=2$. The onset of SEI fracture occurs when $K_{I}$ exceeds $K_{Ic}$.


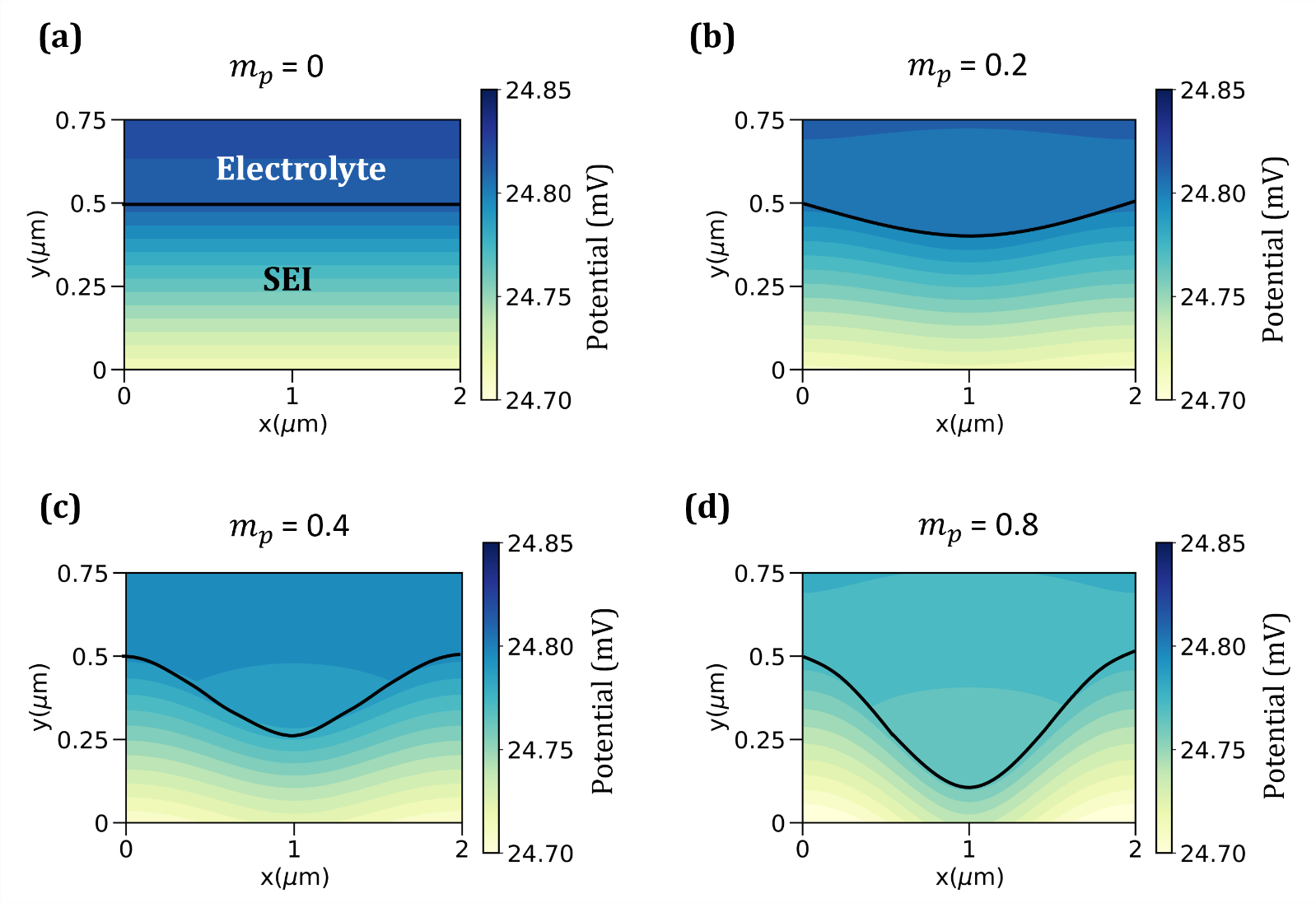


**Figure S1**. Electric potential distribution in the SEI and electrolyte for different morphology parameters, $m_{p}$*=* $D/{L_{SEI}}$: (a) $m_{p}$*=* 0, (b) $m_{p}$*=* 0.2, (c) $m_{p}$*=* 0.4 and (d) $m_{p}$*=* 0.8.


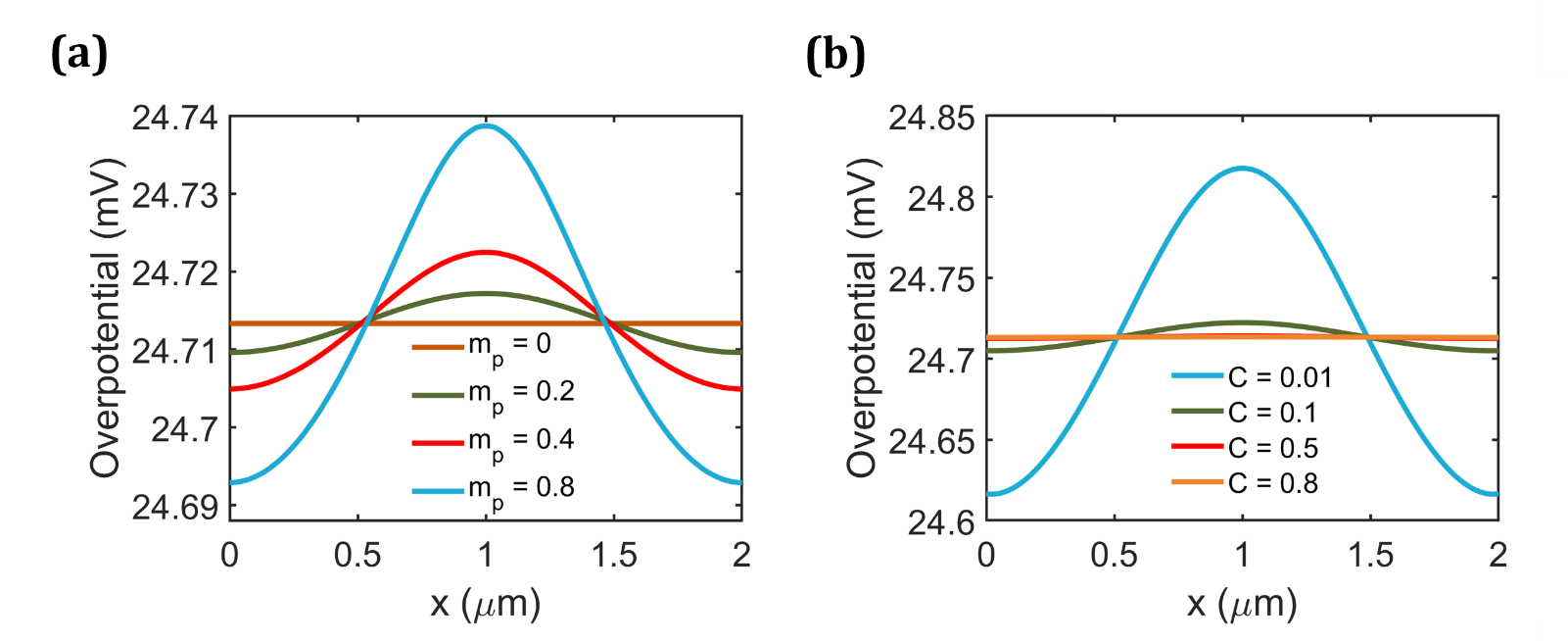


**Figure S2.** Electric overpotential at Na/SEI interface for different values of (a) morphology parameter, $m_{p}$and (b) SEI ionic conductivity $(denoted by C$ *=* ${k_{SEI}}/{k_{elec}})$*.*


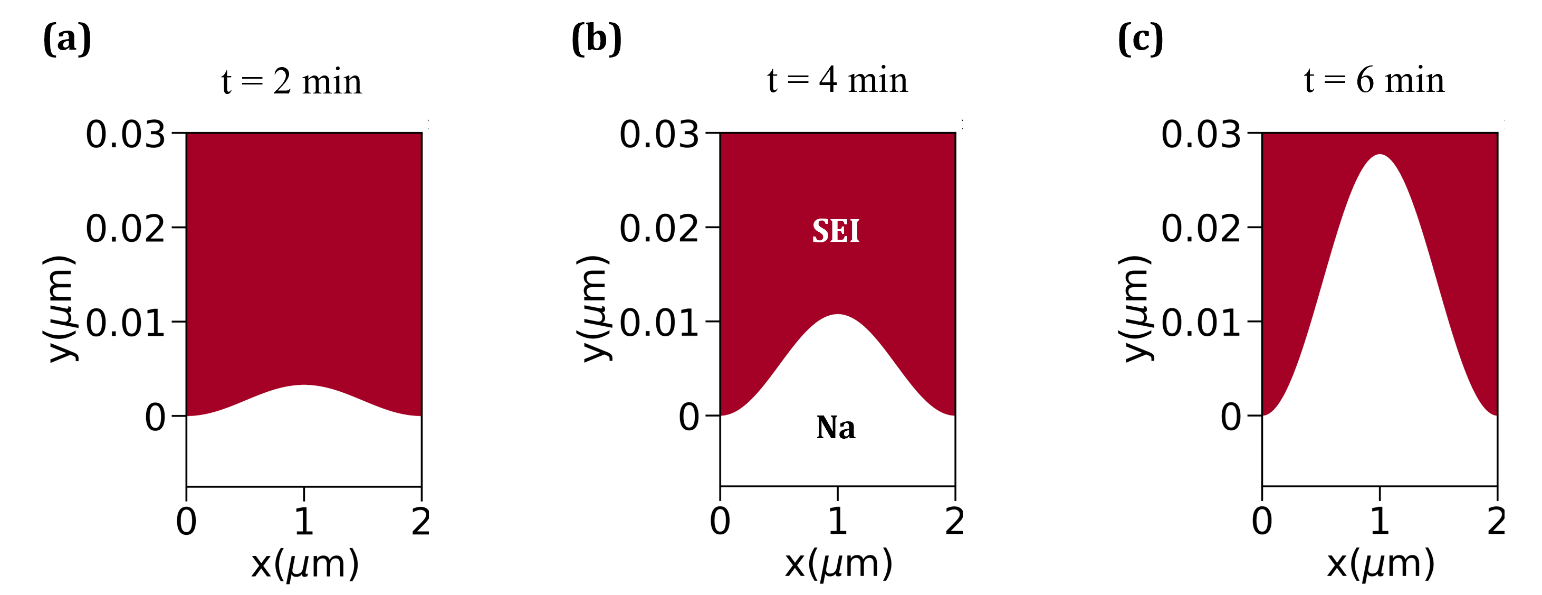


**Figure S3.** Interface morphology at times, $t$ = 2, 4 and 6 minutes. $I_{App}$ = 1 mA cm^-2^, $m_{p}$ = 0.4, ${k_{SEI}}/{k_{elec}}$ = 0.1 and $E_{SEI}$ = 2 GPa.


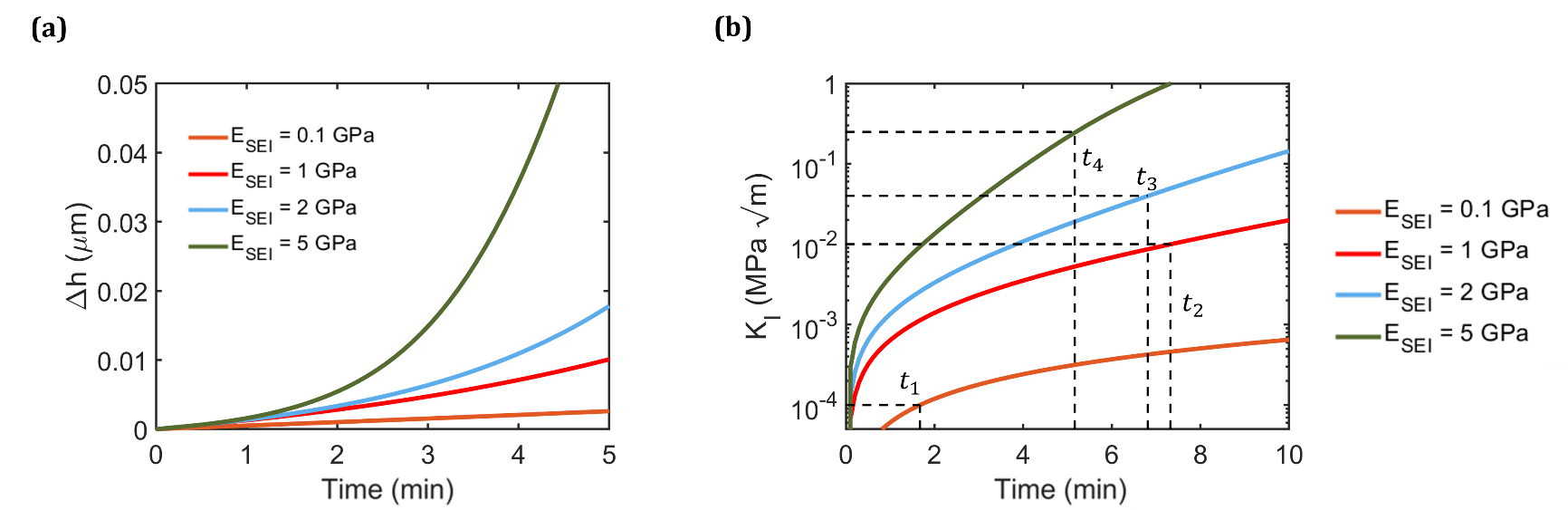


**Figure S4.** Evolution of (a) filament height and (b) Mode I stress intensity ($K_{I})$ for different values of SEI Young’s modulus ($E_{SEI})$.


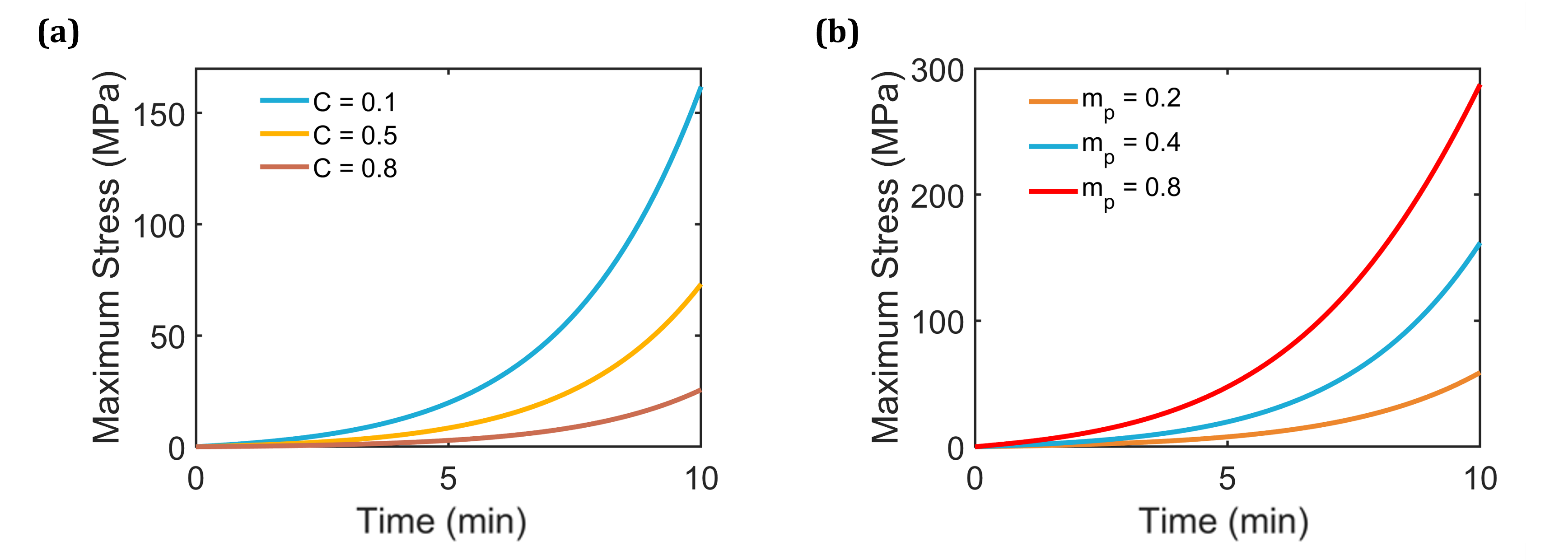


**Figure S5.** Evolution of maximum hydrostatic stress in the SEI for different values of (c) ionic conductivity of SEI $(denoted by C$ *=* ${k_{SEI}}/{k_{elec}})$ and (d) morphology parameter, $m_{p}$.


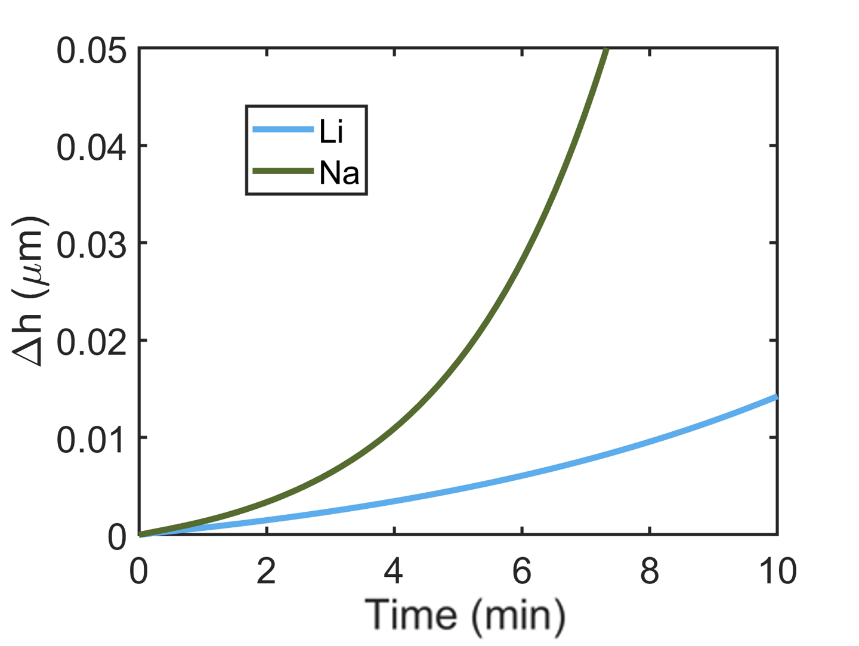


**Figure S6.** Evolution of filament height with time for Na and Li. $I_{App}$ = 1 mA cm^-2^, $m_{p}$ = 0.4.

**References**

[1] W. Liu, P. Liu, D. Mitlin, *Adv Energy Mater* **2020**, *10.*

[2] J. Lee, J. Kim, S. Kim, C. Jo, J. Lee, *Materials Advances* **2020**, *1*, 3143-3166.

[3] M. Mandl, J. Becherer, D. Kramer, R. Mönig, T. Diemant, R. J. Behm, M. Hahn, O. Böse, M. A. Danzer, *Electrochimica Acta* **2020**, *354.*

[4] J. Song, B. Xiao, Y. Lin, K. Xu, X. Li, *Adv Energy Mater* **2018**, *8*.

[5] K. Westman, R. Dugas, P. Jankowski, W. Wieczorek, G. Gachot, M. Morcrette, E. Irisarri, A. Ponrouch, M. R. Palacín, J. M. Tarascon, P. Johansson, *ACS Applied Energy Materials* **2018**, *1*, 2671-2680.

[6] Z. W. Seh, J. Sun, Y. Sun, Y. Cui, *ACS Cent Sci* **2015**, *1*, 449-455.

[7] D. Morales, R. E. Ruther, J. Nanda, S. Greenbaum, *Electrochimica Acta* **2019**, *304*, 239-245.

[8] C. D. Fincher, Y. Zhang, G. M. Pharr, M. Pharr, *ACS Applied Energy Materials* **2020**, *3*, 1759-1767.

[9] P. Barai, K. Higa, V. Srinivasan, *Journal of The Electrochemical Society* **2018**, *165*, A2654-A2666.

[10] D. J. GREEN, *Journal of the American Ceramic Society* **1983**, *66*, 288-292.

[11] C. C. Yuan, X. K. Xi, *Journal of Applied Physics* **2011**, *109.*

[12] K. Guo, R. Kumar, X. Xiao, B. W. Sheldon, H. Gao, *Nano Energy* **2020**, *68*.
